# Qualitative Research: Understanding Patients' Needs and Experiences

**DOI:** 10.1371/journal.pmed.0040258

**Published:** 2007-08-28

**Authors:** The PLoS Medicine Editors

## Abstract

The*PLoS Medicine* Editors argue that the appropriate use of qualitative methods can play an important role in the research process.

Why do up to half of all patients with tuberculosis (TB) fail to adhere to drug treatment [1]? The answer to this question is a matter of life and death, since nonadherence contributes to disease relapse and mortality [2]. In last month's *PLoS Medicine,* Salla Munro and colleagues argue that qualitative studies—in which researchers listen to what patients, care givers, and health care providers have to say—can provide important insights into why nonadherence occurs [3]. Their paper is a “meta-ethnography” [4], a systematic review and synthesis of qualitative studies on adherence to TB medication. The review found a wide array of factors to explain nonadherence, such as the belief that if one's symptoms have disappeared there is no need to finish a course of treatment. We published this review because we thought it would play a role in improving the delivery of TB treatment and ultimately in reducing the enormous global burden of the disease.

*PLoS Medicine* has now published two such meta-ethnographies (the first looked at adherence to HIV medication [5]). We have also published a small number of individual qualitative studies. For example, in our special issue on social medicine (http://collections.plos.org/plosmedicine/socialmedicine-2006.php), we published a qualitative study of migrant workers in the US that found that farm working and housing conditions are organized according to ethnicity and citizenship and that this hierarchy determines health disparities [6]. We have been very selective in our editorial decisions about which qualitative studies to publish. In our decision-making process, we have been guided by two crucial questions.

The first question is whether a qualitative approach was the right way to answer the research question. Quantitative research strives to be objective: human beings, health, and illness are the *objects* of investigation. Such investigation has led to extraordinary biomedical advances—yet patients often fail to reap the benefits because health professionals may not understand how best to deliver them in the context of patients' multifaceted lives. The academic editor of Salla Munro and colleagues' study commented that thinking of TB drugs simply as a “biomedical intervention” without factoring in patients' needs and broader social contexts creates circumstances that increase the likelihood of poor adherence to treatment. Qualitative research is the best way to understand these needs and contexts.

Astrid Fletcher and colleagues, for example, used quantitative methods to objectively determine *who* (in terms of age, sex, and education level) did not use the eye-care services available in India [7]. But they adopted a qualitative approach to answer the question of *why* people did not use these services. David Leon and colleagues, during a quantitative study on hazardous alcohol drinking in Russia, learned that much alcohol was consumed in the form of what were described as “surrogates” [8]. Qualitative research helped to identify what these surrogates were—they included eau de Cologne and over-the-counter medications.

When researchers investigate the experiences of people receiving or failing to receive health care, identify themes in these subjective stories, and integrate these themes into the greater context of human life experience, the results are informative to care providers. The usefulness of these results lies precisely in their subjectivity: the *subjects* are telling us, or we are finding out through more subtle observation, what matters to them.

The results of qualitative research can also help to inform the very process of research itself. Qualitative approaches can help us to understand, for example, why some patients decline to participate in clinical trials [9], or how patients experience the trial process itself. They can even be used to refine or improve a clinical trial in “real time.” In a trial of a computerized decision support tool for patients with atrial fibrillation being considered for anticoagulation treatment, Madeleine Murtagh and colleagues used qualitative evidence in deciding to discontinue one arm of the trial (the intervention in that arm was causing confusion amongst the patients and was unlikely to produce valid data) [10]. When a quantitative study is assessing the effectiveness of a complex multifaceted intervention, qualitative methods can help to tease out why such an intervention works or fails [11]. Qualitative approaches can also help to identify which of many possible research questions should receive priority for investigation, often by asking the research participants themselves. For example, patients with asthma may value easy-to-use inhalers more highly than a new class of drug.

Once it is clear that qualitative methods constitute the right approach for a study submitted to *PLoS Medicine,* the second question is whether the study meets our criteria for rigor and relevance. For a study to be suitable, regardless of the methodology, it should address an important topic in clinical medicine or public health and it should have the potential to transform our understanding of the causes or treatment of disease. In assessing any study, quantitative or qualitative, we are always on the lookout for biases, poorly described methods, and limited generalizability or overinterpretation of the data. In specifically assessing qualitative studies, we additionally wish to be reassured that the researchers used some type of “quality control” in analyzing the data—for example, were the data independently analyzed by at least two researchers and did consistent themes emerge from the data each time?

One characteristic of *PLoS Medicine* is the very broad range of research that we have published to date. We feel that such a range is appropriate for a medical journal, since understanding the complex nature of illness and health care requires a variety of different research approaches. “What is involved is not a crossroads where we have to go left or right,” Martyn Hammersley has argued in a discussion of the false dichotomy between quantitative and qualitative research. “A better analogy is a complex maze where we are repeatedly faced with decisions, and where paths wind back on one another” [12].
